# Systemic immune dysregulation and local barrier failure as predictors of septic shock in acute appendicitis: a large-scale multicenter interpretable machine learning study

**DOI:** 10.3389/fcimb.2026.1935756

**Published:** 2026-10-16

**Authors:** Yunfeng Chen, Yunyun Rui

**Affiliations:** Department of Ultrasound, Nanjing Gaochun People’s Hospital, Nanjing, China

**Keywords:** acute appendicitis, clinical decision support, machine learning, septic shock, systemic immune inflammation index, web-based calculator

## Abstract

**Background:**

The transition from localized acute appendicitis to systemic septic shock is driven by complex immune and metabolic dysregulation combined with anatomical barrier failure. Accurately predicting early hemodynamic deterioration remains clinically challenging. This study aimed to develop an interpretable multimodal machine learning model by integrating systemic immunological profiles with local sonographic characteristics.

**Methods:**

This multicenter cohort study included 3000 patients with acute appendicitis. Following rigorous data reduction using the least absolute shrinkage and selection operator regression, eight supervised machine learning algorithms were constructed. The optimal predictive algorithm was comprehensively evaluated across training, internal testing, and independent external validation cohorts. The SHapley Additive exPlanations framework was utilized to provide transparent model interpretability, and the final algorithm was deployed as a dynamic digital calculator.

**Results:**

Six independent predictors were identified, including the Systemic Immune Inflammation Index, C-reactive protein to albumin ratio, glucose to lymphocyte ratio, Prognostic Nutritional Index, periappendiceal fluid, and the sonographic loss of the submucosal echogenic layer. The Random Forest model demonstrated the highest discriminative performance among the evaluated algorithms, achieving an area under the curve of 0.914. The model’s discrimination, calibration, and decision-analytic performance were consistently evaluated in the internal testing and external validation cohorts. SHAP analysis indicated that systemic inflammatory indices and sonographic features reflecting local disease severity made important contributions to the model’s predicted risk of septic shock. These findings should be interpreted as predictive associations rather than evidence of biological causality or synergy.

**Conclusion:**

This multicenter study developed and externally validated an interpretable machine learning model for early risk stratification of septic shock. The publicly accessible calculator may support individualized risk assessment; however, prospective impact evaluation is required to determine whether its clinical implementation facilitates earlier resuscitation or improves patient outcomes.

## Introduction

Acute appendicitis is one of the most prevalent abdominal surgical emergencies worldwide. While typically uncomplicated and highly curable with timely appendectomy, a critical subset of patients rapidly progresses from localized inflammation to severe systemic infection, ultimately culminating in septic shock (He et al., 2024; Kim et al., 2025). Septic shock represents a life-threatening state of circulatory and cellular metabolism abnormalities driven by a profoundly dysregulated host response to infection (Chen et al., 2021). Crucially, the development of early septic shock prior to or during initial treatment is a catastrophic event that significantly escalates the risk of multiorgan dysfunction syndrome and perioperative mortality. Currently, triaging patients relies heavily on empirical clinical acumen, which often lags behind rapidly evolving hemodynamic deterioration (Hu et al., 2021; Ventoulis et al., 2025). Therefore, identifying patients at high risk of septic shock immediately upon admission is of paramount clinical importance for initiating early aggressive resuscitation and expediting source control.

The transition from localized appendiceal inflammation to systemic septic shock is fundamentally rooted in a complex interplay among immune hyperactivation, systemic inflammation, and host nutritional status (Xu et al., 2022). Traditional single inflammatory biomarkers exhibit limited prognostic capability for early sepsis due to their non-specific nature and susceptibility to minor physiological fluctuations (Drăgoescu et al., 2025). Recently, integrated multidimensional indices reflecting the systemic immune-inflammatory and immuno-nutritional crosstalk have emerged as highly robust prognostic surrogates in various acute infectious diseases (Sun et al., 2021). Rather than merely reflecting acute inflammation, these composite profiles elegantly capture the delicate systemic equilibrium among aggressive hyperinflammation, host immune surveillance, and baseline metabolic reserves.

Furthermore, this systemic immunological cascade is directly triggered by the breakdown of local anatomical barriers (Ranuta et al., 2023; Sobczak et al., 2023; Ma et al., 2024). High-resolution abdominal ultrasound provides crucial non-invasive morphological evidence of this destructive process (Garazzino et al., 2019; Chen et al., 2020; Tullo et al., 2023). Distinct sonographic characteristics of the inflamed appendix can indicate irreversible transmural necrosis, acting as the primary gateway for massive bacterial and endotoxin translocation into the systemic circulation (Sartelli et al., 2017; Michailides et al., 2023). Theoretically, integrating local anatomical imaging features with systemic immunological profiles could yield a comprehensive and multimodal predictive landscape for septic shock in acute appendicitis.

Despite this compelling pathophysiological rationale, existing clinical scoring systems are predominantly designed for basic diagnosis rather than risk-stratifying the trajectory toward severe sepsis (Lin et al., 2025; Stoian et al., 2025). Moreover, traditional linear statistical models frequently fail to capture the highly complex, non-linear, and multicollinear interactions inherent in multidimensional immune-metabolic and imaging data (Li et al., 2025; Marouf et al., 2025). Machine learning algorithms have revolutionized predictive medicine by effectively deciphering these intricate high-dimensional data patterns. However, the broader clinical adoption of advanced machine learning models is frequently hindered by their inherent opacity. The integration of explainable artificial intelligence frameworks bridges this crucial gap, offering transparent and patient-specific interpretability that delineates the exact contribution of each clinical parameter to the predicted risk.

Therefore, this multicenter and large-cohort study aimed to develop and rigorously validate an interpretable multimodal machine learning model for the early prediction of septic shock in patients with acute appendicitis. By seamlessly integrating comprehensive systemic immune-inflammatory profiles with local sonographic characteristics, we sought to construct an accurate risk stratification tool. Furthermore, we aimed to deploy this optimized algorithm as a clinically accessible web-based dynamic calculator, thereby facilitating point-of-care clinical decision-making and personalized pre-emptive therapeutic interventions.

## Materials and methods

### Study design and participants

This retrospective, multicenter cohort study was conducted following the Transparent Reporting of a multivariable prediction model for Individual Prognosis Or Diagnosis (TRIPOD) statement. Patients diagnosed with acute appendicitis between January 1, 2020, and December 31, 2025, were consecutively screened. Patients with missing key laboratory or sonographic data were excluded from the analysis. No imputation was performed, and the final analyses were based on complete cases. The numbers of patients screened, excluded, and included in the primary and external validation cohorts are presented in **Supplementary Figure 1**.

The primary cohort comprised 2000 patients from Nanjing Gaochun People’s Hospital. The primary cohort was stratified according to the septic shock outcome and then randomly partitioned into a training dataset and an internal testing dataset at a 7:3 ratio. This approach preserved a similar outcome distribution in both subsets. The external validation cohort remained independent and was not used for model training or hyperparameter optimization. To rigorously evaluate the model’s spatial generalization capability, an independent external validation cohort of 1, 000 patients treated during the exact same period was established at The Fourth Affiliated Hospital of Soochow University.

The study protocol was approved by the Institutional Review Boards (IRB) of both participating centers. Given the retrospective nature of the study and the utilization of anonymized clinical data, the requirement for informed consent was waived.

### Inclusion and exclusion criteria

The inclusion criteria were defined as follows: (1) adult patients (age ≥≥ 18 years); (2) acute appendicitis confirmed by clinical presentation, laboratory tests, and imaging modalities (ultrasound or computed tomography); and (3) patients initially managed with either standardized conservative treatment or surgical intervention.

Patients were excluded based on the following criteria: (1) missing key laboratory or sonographic data required for model construction; (2) pregnancy; (3) concurrent presence of other acute infectious diseases, severe hematological malignancies, or autoimmune disorders; (4) appendiceal tumors; (5) patients who received immunosuppressive therapy or prolonged systemic corticosteroid treatment prior to admission; and (6) patients who developed septic shock exclusively after surgical intervention (postoperative septic shock).

The prediction index time was defined as the time of hospital admission. Candidate predictors were obtained during the prespecified assessment period after admission and before the onset of septic shock. The onset of septic shock was defined as the earliest documented time at which the patient fulfilled the prespecified diagnostic criteria. Patients who had already met the criteria for septic shock before or at the time of predictor assessment were excluded from the incident prediction analysis. Accordingly, only patients without septic shock at the prediction index time and with predictors measured before septic shock onset were included in the prediction cohort.

### Ultrasound examination protocol and image interpretation

Although the initial diagnosis allowed for computed tomography, all patients included in this study strictly required complete ultrasound data; those with missing sonographic variables were excluded. To ensure cross-center comparability, both centers followed a standardized graded compression ultrasound protocol using 3–5 MHz convex transducers for initial screening and 5–12 MHz linear array transducers for detailed appendiceal evaluation.

Examinations were performed and interpreted by board-certified sonographers with ≥5 years of experience, who were blinded to the patients’ subsequent clinical outcomes. To assess interobserver reproducibility, two blinded radiologists retrospectively re-evaluated a random subset of 200 stored images (100 per center). The inter-rater agreement for the two core sonographic predictors was excellent, yielding Cohen’s kappa values of 0.86 for identifying “loss of the submucosal echogenic layer” and 0.91 for detecting “periappendiceal fluid.”

### Data collection and definition of immune-nutritional profiles

A comprehensive set of 35 baseline variables was systematically extracted from the electronic medical records for each patient, serving as the initial candidate feature pool for the machine learning algorithms. These 35 pre-modeling variables encompassed demographic characteristics (e.g., age, gender, body mass index), vital signs upon admission, detailed appendiceal sonographic morphological features (e.g., outer diameter, periappendiceal fluid, and loss of the submucosal echogenic layer), and extensive preoperative laboratory parameters including complete blood counts, comprehensive metabolic panels, and traditional inflammatory markers.

To deeply quantify the systemic immune-inflammatory and immuno-nutritional status of the patients, four integrated indices were mathematically derived from the initial blood samples obtained within 24 hours of admission (strictly pre-surgery). Rather than analyzing isolated cell counts, these composite indices reflect the complex crosstalk between systemic immunity and metabolic nutrition. Specifically, the Systemic Immune-Inflammation Index (SII) was calculated by multiplying the peripheral platelet count by the absolute neutrophil count, and then dividing by the absolute lymphocyte count (P×N/L). The C-Reactive Protein to Albumin Ratio (CAR) was determined by dividing the serum C-reactive protein concentration (mg/L) by the serum albumin level (g/L). Furthermore, the Prognostic Nutritional Index (PNI) was formulated as the sum of the serum albumin level (g/L) and five times the total peripheral lymphocyte count (10^9^/L). Lastly, the Glucose-to-Lymphocyte Ratio (GLR) was computed as the fasting blood glucose level (mmol/L) divided by the absolute lymphocyte count. All indices were inherently continuous variables and were incorporated into the initial 35-feature pool for subsequent dimensionality reduction and feature selection.

### Outcome definition

The primary clinical endpoint of this study was the occurrence of septic shock following admission for acute appendicitis. To accurately capture the early risk associated with the natural progression of the infection, the endpoint was strictly defined based on the treatment modality: for patients undergoing surgical intervention, only septic shock occurring prior to surgery was considered a positive outcome; for patients receiving conservative management, septic shock occurring at any point during the conservative treatment course was included. Any instances of septic shock that developed after surgical intervention were strictly excluded from the positive outcome group.

In accordance with the Third International Consensus Definitions for Sepsis and Septic Shock (Sepsis-3), septic shock was clinically defined as sepsis accompanied by profound circulatory, cellular, and metabolic abnormalities, specifically requiring vasopressor therapy to maintain a mean arterial pressure (MAP) ≥65 mmHg and having a serum lactate level > 2 mmol/L despite adequate fluid resuscitation (Ko et al., 2023; Orellana et al., 2023; Shime et al., 2025). To evaluate the potential influence of treatment-specific observation windows, a sensitivity analysis was performed using a uniform 48-hour prediction horizon after hospital admission. Septic shock occurring within 48 hours was considered the outcome for all patients, regardless of subsequent treatment strategy.

### Feature selection strategy

To construct a parsimonious and robust predictive model, a two-step feature selection process was implemented on the training dataset. To construct a parsimonious and robust predictive model, a two-step feature selection process was implemented on the training dataset. First, to ensure comparability across parameters with widely differing measurement scales, all continuous variables were Z-score standardized (scaled to a mean of 0 and standard deviation of 1). Next, the Least Absolute Shrinkage and Selection Operator (LASSO) regression model was utilized to address potential high-dimensional multicollinearity among the initial candidate variables. A 10-fold cross-validation approach was applied to determine the optimal penalization parameter (*λ*), minimizing the binomial deviance. Subsequently, the non-zero coefficient variables identified by LASSO were incorporated into a multivariable logistic regression analysis. Variables demonstrating a two-sided *P*-value < 0.05 were defined as independent predictors and retained for final machine learning modeling.

### Development and evaluation of machine learning models

Based on the selected features, eight distinct supervised machine learning algorithms were constructed: Random Forest (RF), eXtreme Gradient Boosting (XGBoost), Support Vector Machine (SVM), Logistic Regression (LR), Decision Tree (DT), K-Nearest Neighbors (KNN), Light Gradient Boosting Machine (LightGBM), and Naive Bayes (NB). Model hyperparameters were optimized using grid search coupled with 10-fold cross-validation in the training set to prevent overfitting.

The discriminative performance of the models was evaluated using the Area Under the Receiver Operating Characteristic (ROC) Curve (AUC). Apparent, cross-validation, and resampling ROC techniques were employed across all three cohorts to ensure stability. Additional classification metrics, including F1-score, Recall, Precision, Negative Predictive Value (NPV), Positive Predictive Value (PPV), Sensitivity, and Specificity, were computed. The optimal model was selected based on comprehensive multidimensional performance.

To assess model calibration, calibration curves were plotted using bootstrapping (1, 000 resamples). The net clinical utility of the selected model was quantified using Decision Curve Analysis (DCA), which estimates the clinical net benefit across a continuum of threshold probabilities compared to default strategies. AUC estimates were reported with 95% confidence intervals. Pairwise comparisons of ROC curves between algorithms were performed using the DeLong test for correlated ROC curves, with two-sided P values reported. The classification threshold was determined using a prespecified probability threshold of 0.5 and was subsequently fixed for evaluation in the internal testing and external validation cohorts. No threshold optimization was performed in the validation cohorts.

Because septic shock occurred in approximately 6% of patients, class imbalance was considered during model development. Inverse-frequency class weights were applied during model training and hyperparameter tuning to reduce the potential influence of the majority class. No synthetic oversampling was performed. Ten-fold cross-validation was conducted using outcome-stratified folds.Model discrimination was evaluated using both the area under the receiver operating characteristic curve (ROC-AUC) and the area under the precision-recall curve (PR-AUC). PR-AUC was included because it provides a more informative assessment of model performance when the positive outcome is relatively uncommon. Additional classification metrics included sensitivity, specificity, positive predictive value, negative predictive value, precision, recall, and F1-score.

Cross-validation was used only within the training cohort for model development and hyperparameter optimization. After the final model was established, it was locked and applied directly to the internal testing and external validation cohorts. The ROC and resampling analyses shown for the validation cohorts were based on predictions from this locked model and were used only to assess performance stability and estimate uncertainty. No model refitting, feature selection, hyperparameter tuning, or recalibration was performed using either validation cohort.

### Model interpretability and web deployment

To demystify the “black-box” nature of the advanced machine learning algorithm, the SHapley Additive exPlanations (SHAP) framework was utilized. SHAP values provided both a global perspective on feature importance ranking and a local interpretation of individual patient predictions through beeswarm and waterfall plots. Finally, the optimized model was deployed as a user-friendly, web-based dynamic calculator using the R shiny package to facilitate point-of-care clinical translation.

### Statistical analysis

Statistical analyses were predominantly performed using R software (version 4.3.0). Continuous variables were assessed for normality before analysis and are presented as mean ± standard deviation or median (interquartile range), as appropriate. For comparisons among the training, internal testing, and external validation cohorts, one-way analysis of variance was used for normally distributed continuous variables, and the Kruskal–Wallis test was used for non-normally distributed continuous variables. Categorical variables are presented as frequencies and percentages and were compared using the chi-square test or Fisher’s exact test, as appropriate. For comparisons between two groups, the Student’s *t*-test or Mann–Whitney *U* test was used according to data distribution. All tests were two-sided, and a *P* value < 0.05 was considered statistically significant. Spearman’s rank correlation and Variance Inflation Factor (VIF) analyses were conducted to evaluate multicollinearity, with a VIF < 5 considered acceptable. A two-sided *P*-value < 0.05 was considered statistically significant. In the sensitivity analysis, the model was re-evaluated using septic shock within 48 hours after admission as the outcome. The same modeling strategy was applied, and model performance was assessed in the training, internal testing, and external validation cohorts.

## Result

### Baseline characteristics of the study population

In the primary center, 2, 248 patients were screened. Of these, 248 were excluded, including 96 with missing key laboratory data and 72 with missing key sonographic data. The remaining 2, 000 patients comprised the primary cohort, which was divided into a training cohort (n = 1, 400) and an internal testing cohort (n = 600). In the external center, 1, 137 patients were screened, of whom 137 were excluded. The remaining 1, 000 patients constituted the external validation cohort. The detailed participant selection process and exclusion reasons are shown in **Supplementary Figure 1**. The baseline demographic and clinical characteristics across the three cohorts are detailed in **Table 1** and **Table 2**. Statistical analyses revealed no significant differences among the cohorts regarding age, gender, body mass index (BMI), and conventional laboratory parameters such as white blood cell (WBC) count and C-reactive protein (CRP) (all *P* > 0.05). Furthermore, the overall incidence of septic shock remained consistent across the training, testing, and validation sets, indicating comparable baseline characteristics and a similar septic shock prevalence across the three cohorts.

**Table 1:** Baseline characteristics of patients in the training, testing, and validation cohorts.

Characteristic	Training cohort (n = 1400)	Testing cohort (n = 600)	Validation cohort (n = 1000)	P value
Demographics and clinical presentation				
Age (years), Mean ± SD	58.4 ± 14.2	60.1 ± 13.5	57.6 ± 14.8	0.074
Gender (Male), n (%)	742 (53.0%)	306 (51.0%)	552 (55.2%)	0.218
BMI (kg/m²), Mean ± SD	24.5 ± 3.6	24.1 ± 3.1	24.8 ± 3.9	0.089
Duration of symptoms (hours), Mean ± SD	36.5 ± 18.4	38.2 ± 17.1	35.1 ± 19.6	0.105
Admission heart rate (bpm), Mean ± SD	92.4 ± 16.5	94.1 ± 15.8	91.5 ± 17.2	0.063
Admission SBP (mmHg), Mean ± SD	128.4 ± 18.1	130.5 ± 17.6	127.2 ± 19.5	0.124
Admission DBP (mmHg), Mean ± SD	78.5 ± 11.3	79.8 ± 10.5	77.4 ± 12.1	0.141
Respiratory rate (breaths/min), Mean ± SD	19.5 ± 3.2	20.1 ± 2.9	19.2 ± 3.5	0.058
Body temperature (°C), Mean ± SD	38.1 ± 0.7	38.2 ± 0.6	38.0 ± 0.8	0.082
Medical history				
History of hypertension (Yes), n (%)	462 (33.0%)	216 (36.0%)	310 (31.0%)	0.098
History of diabetes mellitus (Yes), n (%)	266 (19.0%)	134 (22.3%)	175 (17.5%)	0.067
Cardiovascular disease (Yes), n (%)	154 (11.0%)	78 (13.0%)	95 (9.5%)	0.112
Chronic kidney disease (Yes), n (%)	84 (6.0%)	46 (7.7%)	51 (5.1%)	0.145
Previous abdominal surgery (Yes), n (%)	210 (15.0%)	102 (17.0%)	145 (14.5%)	0.363
Smoking history (Yes), n (%)	392 (28.0%)	186 (31.0%)	265 (26.5%)	0.124
Laboratory findings				
WBC count (×10^9/L), Mean ± SD	14.2 ± 4.5	14.8 ± 4.2	13.9 ± 4.8	0.068
Neutrophil count (×10^9/L), Mean ± SD	11.8 ± 3.9	12.4 ± 3.6	11.5 ± 4.1	0.071
Lymphocyte count (×10^9/L), Mean ± SD	1.15 ± 0.45	1.10 ± 0.41	1.18 ± 0.48	0.134
Platelet count (×10^9/L), Mean ± SD	235 ± 65	228 ± 62	241 ± 68	0.187
Random blood glucose (mmol/L), Mean ± SD	8.6 ± 3.2	9.1 ± 3.5	8.4 ± 3.0	0.059
CRP (mg/L), Mean ± SD	65.4 ± 28.5	68.2 ± 26.1	63.5 ± 30.2	0.091
Serum albumin (g/L), Mean ± SD	38.5 ± 5.4	37.8 ± 5.1	39.1 ± 5.8	0.076
Serum creatinine (μmol/L), Mean ± SD	85.4 ± 22.1	88.5 ± 21.4	83.2 ± 24.5	0.088
Serum lactate (mmol/L), Mean ± SD	1.8 ± 0.6	1.9 ± 0.5	1.7 ± 0.7	0.054
Systemic immune & metabolic indices				
SII, Mean ± SD	2450 ± 1120	2610 ± 1080	2380 ± 1180	0.065
CAR, Mean ± SD	1.75 ± 0.85	1.84 ± 0.81	1.68 ± 0.92	0.079
PNI, Mean ± SD	44.2 ± 6.5	43.3 ± 6.1	45.0 ± 6.8	0.083
GLR, Mean ± SD	8.4 ± 3.8	9.0 ± 3.5	8.1 ± 4.1	0.061
Clinical Scoring				
Alvarado Score, Mean ± SD	6.8 ± 1.5	7.0 ± 1.4	6.6 ± 1.6	0.072
qSOFA score ≥ 2 (Yes), n (%)	112 (8.0%)	58 (9.7%)	72 (7.2%)	0.155
Ultrasound (US) characteristics				
Appendiceal outer diameter (mm), Mean ± SD	9.5 ± 2.8	9.8 ± 2.5	9.2 ± 3.1	0.066
Loss of submucosal echogenic layer (Yes), n (%)	252 (18.0%)	126 (21.0%)	165 (16.5%)	0.081
Periappendiceal fluid (Yes), n (%)	392 (28.0%)	186 (31.0%)	265 (26.5%)	0.124
Presence of appendicolith (Yes), n (%)	448 (32.0%)	210 (35.0%)	295 (29.5%)	0.118
Increased periappendiceal echogenicity (Yes), n (%)	658 (47.0%)	306 (51.0%)	445 (44.5%)	0.069
Outcome				
Septic shock (Yes), n (%)	84 (6.00%)	37 (6.17%)	58 (5.80%)	0.912

Data are presented as mean ± standard deviation (SD) for normally distributed continuous variables, and as number (percentage) for categorical variables. P values indicate the overall differences among the training, testing, and validation cohorts, calculated using analysis of variance (ANOVA) for continuous variables and the Pearson’s Chi-square test for categorical variables.

SD, standard deviation; BMI, body mass index; SBP, systolic blood pressure; DBP, diastolic blood pressure; WBC, white blood cell; CRP, C-reactive protein; SII, Systemic Immune-Inflammation Index; CAR, C-Reactive Protein to Albumin Ratio; PNI, Prognostic Nutritional Index; GLR, Glucose-to-Lymphocyte Ratio; qSOFA, quick Sequential Organ Failure Assessment.

**Table 2:** Variable assignments.

Variables	Risk factors	Assignment
X1	Age	Continuous variable
X2	Gender	Male=1, Female=0
X3	BMI	Continuous variable
X4	Duration of symptoms	Continuous variable
X5	Admission heart rate	Continuous variable
X6	Admission SBP	Continuous variable
X7	Admission DBP	Continuous variable
X8	Respiratory rate	Continuous variable
X9	Body temperature	Continuous variable
X10	History of hypertension	Yes=1, No=0
X11	History of diabetes mellitus	Yes=1, No=0
X12	Cardiovascular disease	Yes=1, No=0
X13	Chronic kidney disease	Yes=1, No=0
X14	Previous abdominal surgery	Yes=1, No=0
X15	Smoking history	Yes=1, No=0
X16	WBC count	Continuous variable
X17	Neutrophil count	Continuous variable
X18	Lymphocyte count	Continuous variable
X19	Platelet count	Continuous variable
X20	Random blood glucose	Continuous variable
X21	CRP	Continuous variable
X22	Serum albumin	Continuous variable
X23	Serum creatinine	Continuous variable
X24	Serum lactate	Continuous variable
X25	SII	Continuous variable
X26	CAR	Continuous variable
X27	PNI	Continuous variable
X28	GLR	Continuous variable
X29	Alvarado Score	Continuous variable
X30	qSOFA score ≥ 2	Yes=1, No=0
X31	Appendiceal outer diameter	Continuous variable
X32	Loss of submucosal echogenic layer	Yes=1, No=0
X33	Periappendiceal fluid	Yes=1, No=0
X34	Presence of appendicolith	Yes=1, No=0
X35	Increased periappendiceal echogenicity	Yes=1, No=0
Y	Septic shock (Outcome)	Yes=1, No=0

Continuous variables were directly inputted as numerical values into the machine learning algorithms. Binary categorical variables were encoded using one-hot encoding (assigned as 1 for presence and 0 for absence). The composite immune-metabolic indices were calculated as follows: SII = (Platelet count × Neutrophil count)/Lymphocyte count; CAR = CRP/Serum albumin; PNI = Serum albumin (g/L) + 5 × Lymphocyte count (×10^9/L); GLR = Blood glucose (mmol/L)/Lymphocyte count (×10^9/L).

BMI, body mass index; SBP, systolic blood pressure; DBP, diastolic blood pressure; WBC, white blood cell; CRP, C-reactive protein; SII, Systemic Immune-Inflammation Index; CAR, C-Reactive Protein to Albumin Ratio; PNI, Prognostic Nutritional Index; GLR, Glucose-to-Lymphocyte Ratio; qSOFA, quick Sequential Organ Failure Assessment.

### Feature selection and identification of immune-nutritional risk factors

Given the high collinearity observed among raw, individual inflammatory markers in the initial candidate pool, we employed the Least Absolute Shrinkage and Selection Operator (LASSO) regression with 10-fold cross-validation to perform rigorous dimensionality reduction (**Figures 1A, B**). Subsequently, multivariable logistic regression analysis was conducted, identifying six independent predictors for septic shock. Feature selection was performed exclusively within the training cohort. LASSO regression was first used to identify candidate predictors, followed by multivariable logistic regression to determine the final variables used for model development. The internal testing and external validation cohorts were not involved in feature selection or model training. Cross-validation was conducted within the training cohort to minimize information leakage and overestimation of model performance.

**Figure 1:**
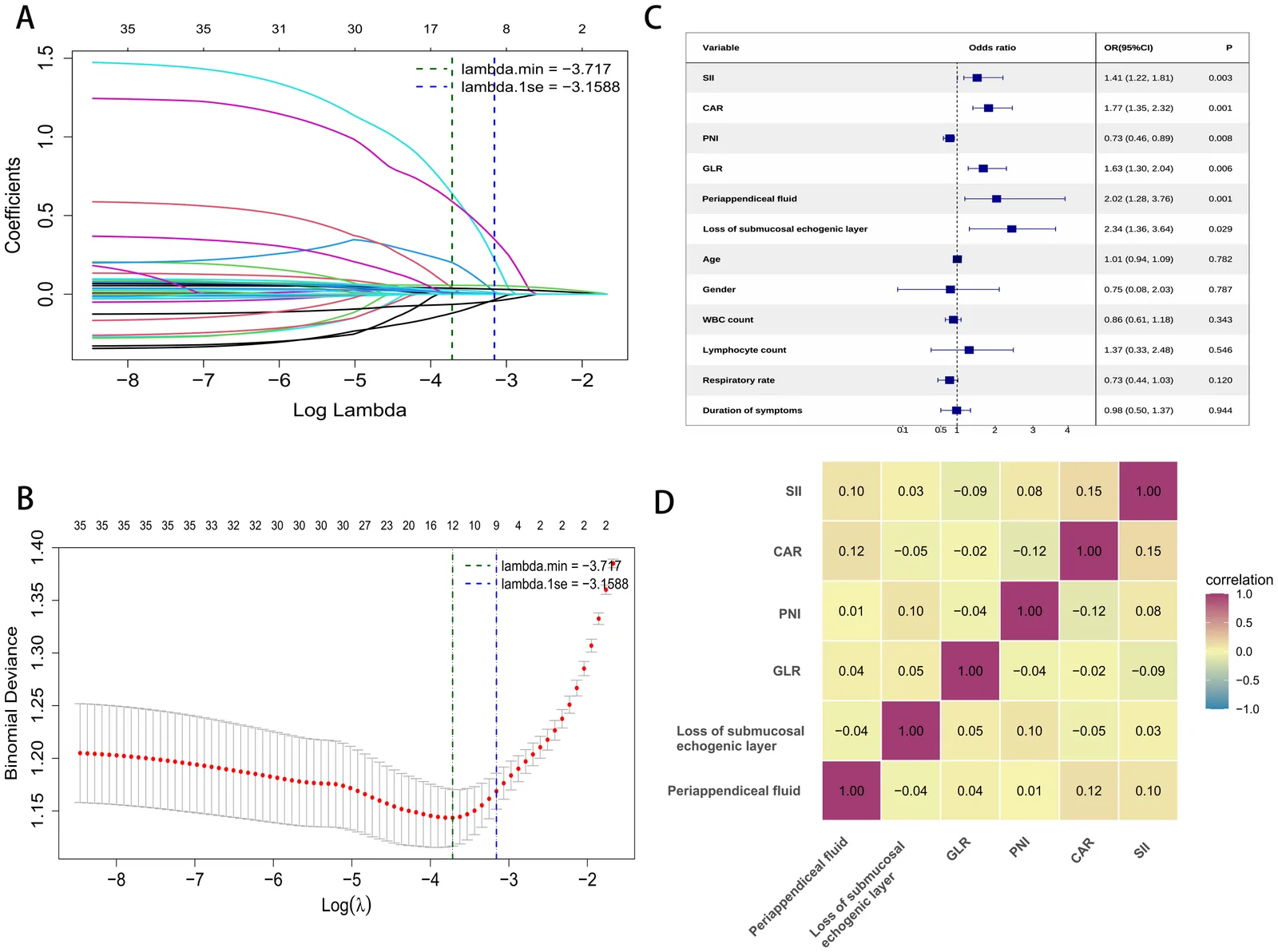
Feature selection and identification of independent risk factors. **(A, B)** Least Absolute Shrinkage and Selection Operator (LASSO) regression used for dimensional reduction, displaying the coefficient profile plot and the tuning parameter (λ) selection via 10-fold cross-validation. **(C)** Forest plot summarizing the multivariable logistic regression results, with Odds Ratios (OR) and 95% Confidence Intervals indicating the independent predictive value of the selected features. For continuous variables (SII, CAR, GLR, and PNI), the Odds Ratios (OR) represent the risk change per 1-standard deviation (SD) increase. **(D)** A correlation heatmap illustrating the inter-variable relationships among the six final predictors, generated using Spearman’s rank correlation analysis. The low correlation coefficients confirm the absence of substantial multicollinearity, ensuring model stability.

Notably, systemic immune-inflammatory and immuno-nutritional indices emerged as critical factors. The multivariable forest plot (**Figure 1C**) confirmed that the Systemic Immune-Inflammation Index (SII; OR per 1-SD increase = 1.41, 95% CI: 1.22–1.81, *P* = 0.003), C-Reactive Protein to Albumin Ratio (CAR; OR per 1-SD increase= 1.77, 95% CI: 1.35–2.32, *P* = 0.001), and Glucose-to-Lymphocyte Ratio (GLR; OR per 1-SD increase = 1.63, 95% CI: 1.30–2.04, *P* = 0.006) were independent risk factors. Conversely, a higher Prognostic Nutritional Index (PNI) served as a significant protective factor (OR per 1-SD increase = 0.73, 95% CI: 0.46–0.89, *P* = 0.008). Two ultrasound features, periappendiceal fluid and the loss of the submucosal echogenic layer, were also independently associated with higher risk. Spearman’s rank correlation analysis (**Figure 1D**) demonstrated minimal correlation coefficients (maximum r=0.15) among these six final predictors. Variance inflation factors (VIF) were all strictly below 2.0 (**Table 3**), successfully minimizing information redundancy and ensuring the stability of the subsequent machine learning models.

**Table 3:** Collinearity analysis of candidate and selected variables.

Variable	VIF (all candidate variables)	VIF (selected features)	Inclusion in final model
Age	1.450392847		
Gender	1.182938475		
BMI	1.347201938		
Duration of symptoms	1.150938475		
Admission heart rate	2.140392847		
Admission SBP	4.620938475		
Admission DBP	4.140392847		
Respiratory rate	1.620394857		
Body temperature	1.852839472		
History of hypertension	1.450394857		
History of diabetes mellitus	1.652839472		
Cardiovascular disease	1.324750293		
Chronic kidney disease	1.215039284		
Previous abdominal surgery	1.120394857		
Smoking history	1.347201938		
WBC count	6.950392847		
Neutrophil count	7.847201938		
Lymphocyte count	9.232930485		
Platelet count	3.118201938		
Random blood glucose	4.193847562		
CRP	6.842750293		
Serum albumin	7.270394857		
Serum creatinine	1.310938475		
Serum lactate	1.458374629		
SII	8.847201938	1.352837461	Yes
CAR	6.120394857	1.284029384	Yes
PNI	7.650392847	1.410394857	Yes
GLR	5.882930485	1.154750293	Yes
Alvarado Score	3.550392847		
qSOFA score ≥ 2	2.630938475		
Appendiceal outer diameter	1.847201938		
Loss of submucosal echogenic layer	1.623948572	1.082938475	Yes
Periappendiceal fluid	1.750392847	1.124750293	Yes
Presence of appendicolith	1.200394857		
Increased periappendiceal echogenicity	1.892938475		

Data demonstrates that while raw inflammatory cells and biochemical markers showed strong multicollinearity (VIF > 5) in the initial candidate pool due to their mathematical coupling, the final 6 selected immuno-sonographic features exhibited VIF values well below 2, indicating complete independence and minimal redundancy in the optimal predictive model.

VIF, variance inflation factor; BMI, body mass index; SBP, systolic blood pressure; DBP, diastolic blood pressure; WBC, white blood cell; CRP, C-reactive protein; SII, Systemic Immune-Inflammation Index; CAR, C-Reactive Protein to Albumin Ratio; PNI, Prognostic Nutritional Index; GLR, Glucose-to-Lymphocyte Ratio; qSOFA, quick Sequential Organ Failure Assessment.

### Construction and evaluation of machine learning models

To identify the optimal predictive algorithm, eight supervised machine learning models (Random Forest [RF], XGBoost, Support Vector Machine [SVM], Logistic Regression [LR], Decision Tree [DT], K-Nearest Neighbors [KNN], LightGBM, and Naive Bayes) were constructed using the six selected variables. In the training set, the RF model exhibited the most outstanding discriminative performance, achieving the highest Area Under the Curve (AUC) of 0.914 (**Figure 2A**).

**Figure 2:**
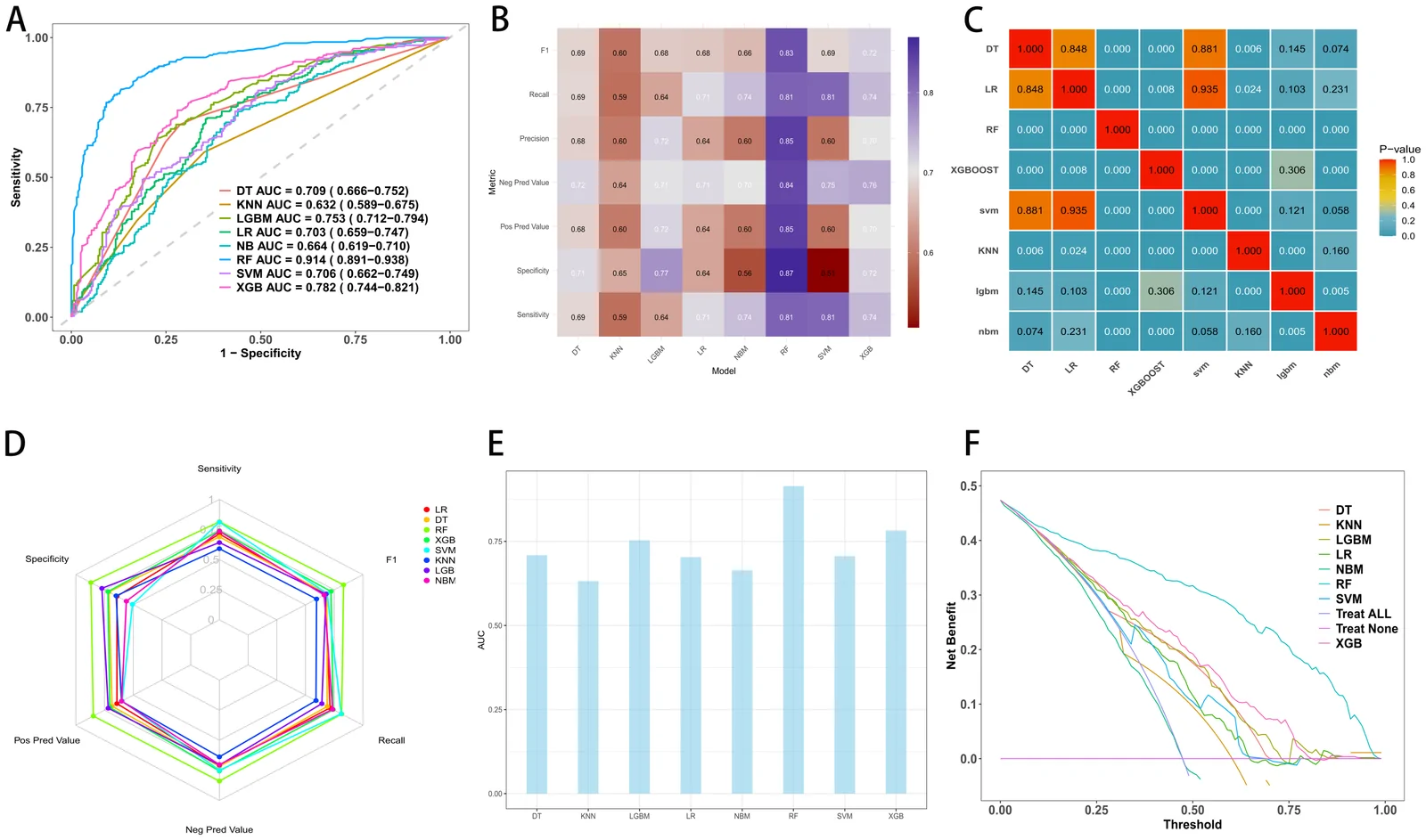
Performance comparison of eight machine learning models. **(A)** Receiver operating characteristic (ROC) curves and corresponding Area Under the Curve (AUC) values for the models. The Random Forest (RF) model exhibited the highest AUC (0.914). **(B)** Heatmap summarizing comprehensive evaluation metrics (F1-score, recall, precision, NPV, PPV, specificity, and sensitivity). **(C)** Matrix of statistical significance (P-values) for pairwise model comparisons. **(D, E)** Radar chart and bar plot visualizing multidimensional performance metrics. **(F)** Decision curve analysis (DCA) demonstrating the net clinical benefit of different algorithms across threshold probabilities.

A comprehensive, multidimensional performance evaluation further highlighted the superiority of the RF algorithm. As depicted in the metric heatmap and radar chart (**Figures 2B, D**), the RF model outperformed others in terms of F1-score, recall, precision, and specificity. The pairwise statistical matrix (**Figure 2C**) confirmed the significant advantage of RF over weaker learners like DT and KNN. Furthermore, Decision Curve Analysis (DCA) indicated that the RF model provided the maximum net clinical benefit across nearly all threshold probabilities compared to alternative algorithms (**Figure 2F**). Consequently, RF was selected as the final model for further multidimensional validation.

### Multidimensional validation of the optimal model

To rigorously assess the model’s stability and prevent overfitting, a three-tiered validation strategy incorporating apparent, cross-validation, and resampling ROC curves was employed (**Figure 3**). In the training cohort, the RF model maintained robust predictive accuracy across all ROC testing variations (**Figures 3A–C**).

**Figure 3:**
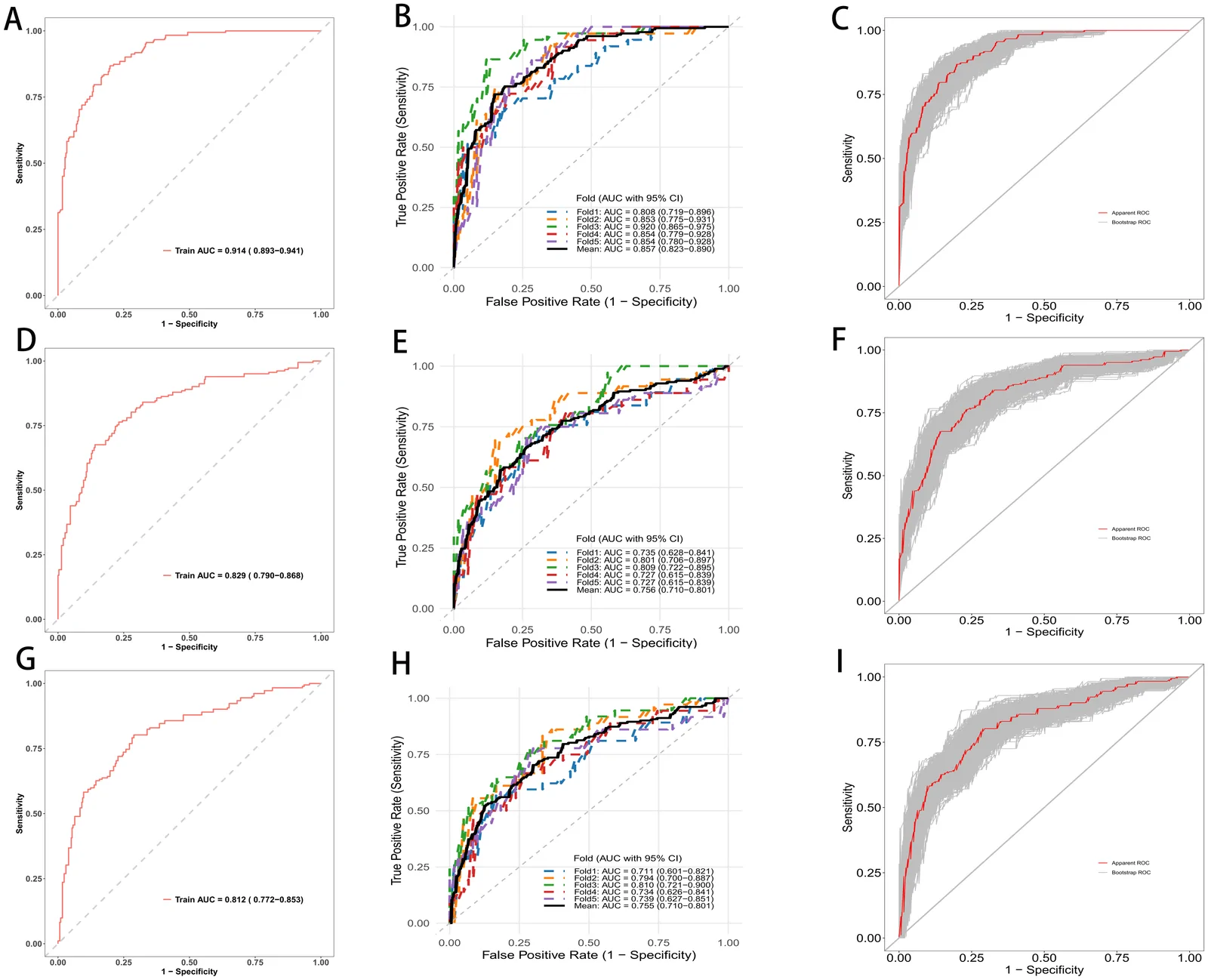
Comprehensive evaluation of model discrimination and stability across three independent cohorts. The diagnostic performance was rigorously assessed using standard ROC curves, alongside cross-validation and resampling techniques to prevent overfitting. Panels **(A–C)** illustrate the model’s predictive accuracy within the training set (n=1400), representing the apparent ROC, cross-validation ROC, and resampling ROC curves, respectively. To confirm internal validity, panels **(D–F)** detail the corresponding ROC analyses (apparent, cross-validation, and resampling) for the internal testing set (n=600). Furthermore, panels **(G–I)** demonstrate the model’s robust generalization capability (apparent, cross-validation, and resampling ROCs) when applied to the external validation set (n=1000). The consistently high AUC across these varying validation strategies highlights the model’s reliability in predicting septic shock.

Crucially, this high discriminative ability was successfully replicated in the unseen data. In the internal testing cohort (n=600), the model demonstrated sustained performance without substantial performance degradation (**Figures 3D–F**). More importantly, when applied to the completely independent external validation cohort (n=1000), the RF model yielded highly consistent AUC values (**Figures 3G–I**). The consistent performance in the external validation cohort supported the model’s transportability within the settings evaluated in this study.In the sensitivity analysis using a uniform 48-hour prediction horizon, the Random Forest model showed consistent discrimination across the three cohorts. The AUCs were 0.902 in the training cohort, 0.886 in the internal testing cohort, and 0.878 in the external validation cohort (**Supplementary Table 1**).

Because septic shock was relatively uncommon, model performance was additionally assessed using the area under the precision-recall curve. For the Random Forest model, the ROC-AUC and PR-AUC were 0.914 and 0.421, respectively, in the training cohort, 0.829 and 0.356, respectively, in the internal testing cohort, and 0.812 and 0.331, respectively, in the external validation cohort. These findings supported the discriminatory ability of the model after accounting for the relatively low prevalence of septic shock(**Supplementary Table 2**).

### Calibration and clinical utility assessment

The practical applicability of the RF model was evaluated using calibration curves and decision curve analysis (DCA) across all three cohorts (**Figure 4**). The calibration plots in the training (**Figure 4A**), testing (**Figure 4D**), and external validation (**Figure 4G**) sets all closely tracked the ideal 45-degree reference line and showed generally good agreement between predicted and observed risks across the evaluated cohorts. Calibration was assessed using calibration plots, calibration intercepts, calibration slopes, and Brier scores. The calibration intercepts were close to zero in the training cohort (0.00, 95% CI: -0.26 to 0.26), internal testing cohort (-0.00, 95% CI: -0.22 to 0.22), and external validation cohort (-0.00, 95% CI: -0.22 to 0.22). The corresponding calibration slopes were 1.00 (95% CI: 0.82 to 1.18), 1.00 (95% CI: 0.80 to 1.20), and 1.00 (95% CI: 0.80 to 1.20), respectively. The Brier scores were 0.156 (95% CI: 0.134 to 0.167), 0.196 (95% CI: 0.178 to 0.221), and 0.183 (95% CI: 0.146 to 0.287), respectively(**Supplementary Table 3**). Overall, these findings suggested generally good agreement between predicted and observed risks across the three cohorts, although the wider confidence interval for the Brier score in the external validation cohort indicated greater uncertainty in the calibration estimate.Additionally, confusion matrices visualized the specific classification accuracy across cohorts (**Figures 4B, E, H**).

**Figure 4:**
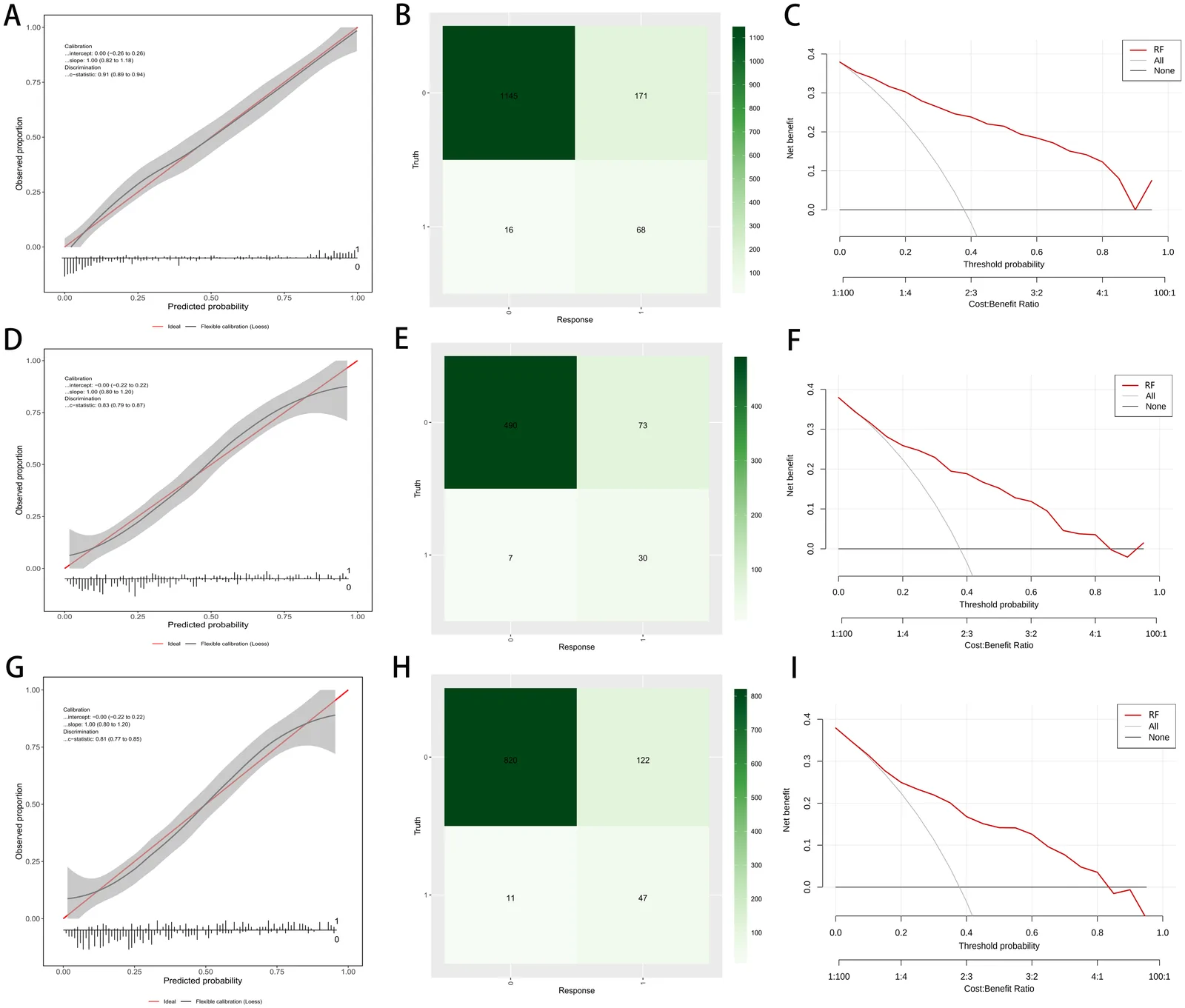
Assessment of model calibration, classification metrics, and net clinical utility. For each cohort, a combination of calibration plots, confusion matrices, and Decision Curve Analysis (DCA) was utilized. Findings derived from the training dataset are presented in **(A–C)**, corresponding to the calibration curve, confusion matrix, and DCA, respectively. This is followed by results validating the model’s predictive agreement in the internal testing set **(D–F)** (calibration, confusion matrix, and DCA, respectively), and its sustained performance within the external validation cohort **(G–I)**. The confusion matrices detail the specific classification accuracy, and the DCA curves confirm that deploying this predictive tool yields a superior net clinical benefit compared to default strategies (treat-all or treat-none) across a broad spectrum of threshold probabilities.

The DCA curves consistently demonstrated that utilizing the RF model to guide clinical interventions yields a superior net clinical benefit compared to default “treat-all” or “treat-none” strategies across a broad spectrum of threshold probabilities in both internal and external cohorts (**Figures 4C, F, I**).

### Global and local interpretability via SHAP values

To elucidate the decision-making process of the optimal RF model, SHapley Additive exPlanations (SHAP) were employed for comprehensive global and local interpretability. The SHAP feature importance analysis (**Figure 5A**) identified the systemic immune-inflammatory biomarker, SII, as the paramount contributor to the predictive model, followed by the loss of the submucosal echogenic layer, CAR, GLR, and PNI.

**Figure 5:**
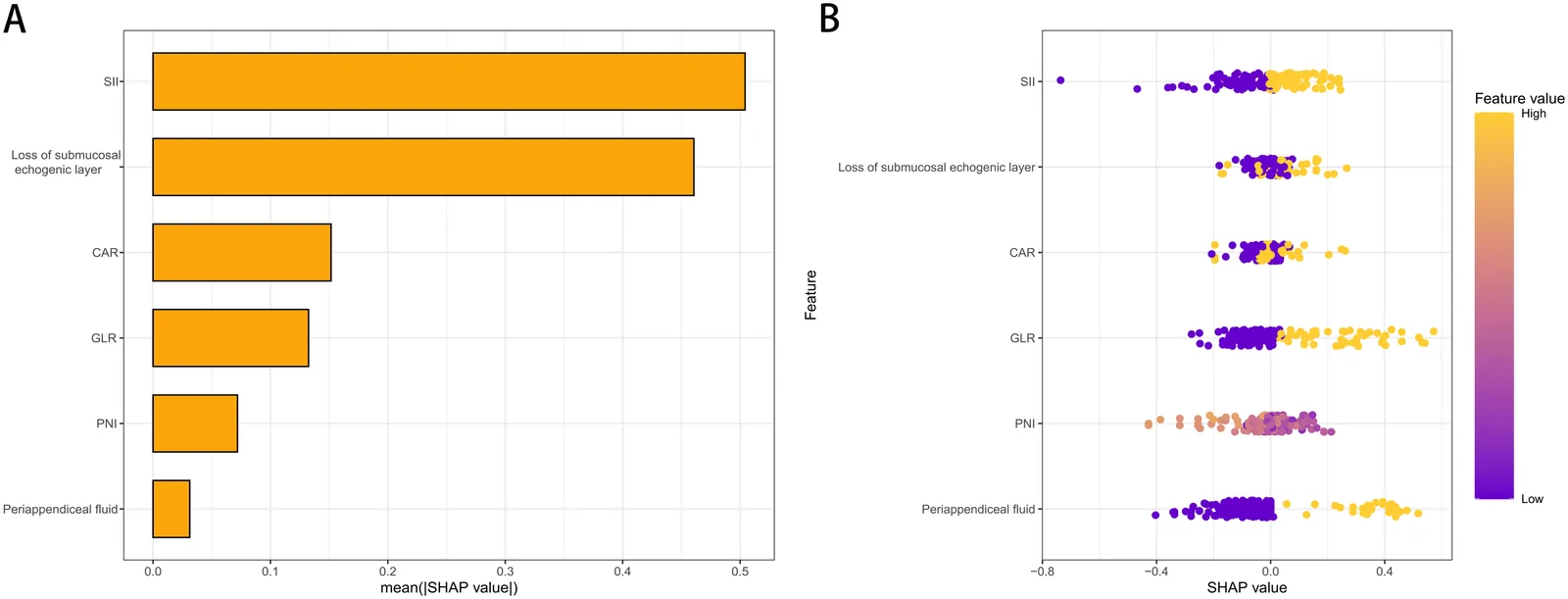
Global interpretability of the prediction model using SHapley Additive exPlanations (SHAP). **(A)** SHAP feature importance bar plot ranking the overall contribution of the six predictors to the model’s output. **(B)** SHAP summary beeswarm plot illustrating the distribution of SHAP values. The color gradient (from purple to yellow) represents the feature values from low to high, demonstrating the directional impact (risk-increasing or risk-decreasing) of each feature on the probability of septic shock.

The SHAP summary beeswarm plot (**Figure 5B**) delineated the directional impact of these predictors on the model’s output. Specifically, the combination of an elevated systemic inflammatory profile, characterized by high SII, CAR, and GLR, and local anatomical destruction (ultrasound signs of submucosal layer loss) significantly increased the predicted probability of septic shock. Conversely, a robust immuno-nutritional status, as indicated by higher PNI values, exhibited a substantial protective effect.

To facilitate point-of-care clinical translation, the established RF model was deployed as a publicly accessible, web-based dynamic calculator (available at: https://medicalinformaticsshinyposter.shinyapps.io/shock-infection-predictor/). This interactive platform enables clinicians to input individualized patient profiles and obtain a real-time probability of septic shock. Furthermore, the interface generates a patient-specific SHAP waterfall plot, quantitatively detailing the exact contribution of each clinical and sonographic feature to the final prediction. **Figure 6** illustrates two representative clinical scenarios evaluated by this calculator: a low-risk patient with a predicted septic shock probability of 2.0% (**Figure 6A**) and a high-risk patient with a predicted probability of 59.0% (**Figure 6B**), demonstrating the tool’s robust capacity for personalized risk stratification. SHAP analysis showed that systemic inflammatory markers and indicators of local barrier disruption contributed substantially to the model’s predictions of septic shock risk. These findings suggest that both systemic inflammatory status and local disease-related features may be important for risk stratification. However, SHAP values reflect the contribution of predictors to model predictions and do not establish causality or statistical interaction between predictors.

**Figure 6:**
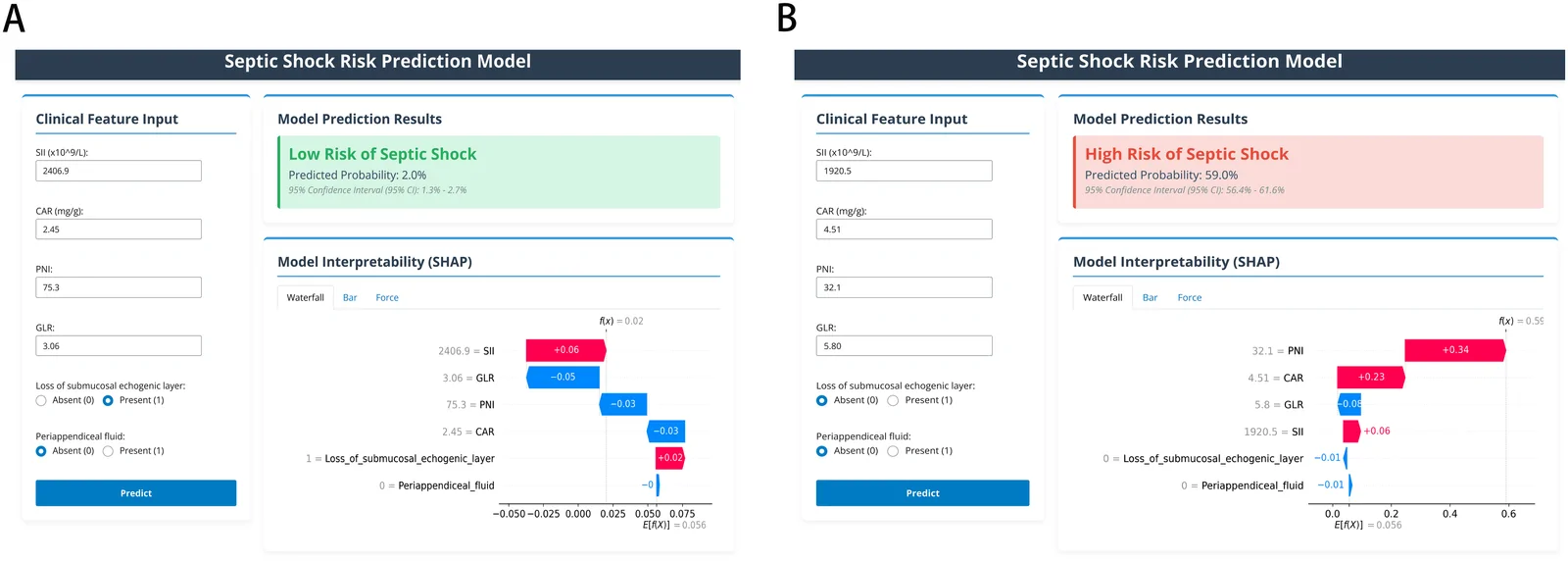
Web-based dynamic calculator and individualized prediction. **(A)** Interface output for a representative low-risk patient (predicted probability: 2.0%), accompanied by the corresponding SHAP waterfall plot detailing individual feature contributions. **(B)** Interface output for a representative high-risk patient (predicted probability: 59.0%), accompanied by the SHAP waterfall plot. Red bars indicate features pushing the prediction higher, while blue bars indicate features pushing the prediction lower.

### Incremental value over conventional clinical scores

To determine whether the inclusion of immune-nutritional and ultrasound variables provided material incremental value, the optimal Random Forest model was compared directly with established clinical baseline models (qSOFA and Alvarado scores). In terms of discrimination, the Random Forest model exhibited significantly higher ROC-AUCs than both qSOFA (AUC: 0.671, 95% CI: 0.608–0.734; DeLong test P < 0.001) and Alvarado scores (AUC: 0.575, 95% CI: 0.510–0.640; DeLong test P < 0.001) in the external validation cohort. Similar highly significant improvements were consistently observed in the training and internal testing cohorts.

Regarding calibration, the Random Forest model demonstrated more precise absolute risk estimation, yielding consistently lower Brier scores across all cohorts compared to the conventional models. In the external validation cohort, the Random Forest model achieved a superior Brier score of 0.183, compared to 0.268 for qSOFA and 0.291 for the Alvarado score (**Supplementary Table 4**). Furthermore, Decision Curve Analysis empirically confirmed that the Random Forest model provided a substantially higher net clinical benefit than evaluating patient risk using qSOFA or Alvarado scores alone(**Supplementary Figure 2**).

## Discussion

In this large-scale multicenter cohort study encompassing 3, 000 patients, we successfully developed and externally validated a highly accurate, interpretable machine learning framework for the early prediction of preoperative septic shock in patients with acute appendicitis. Through rigorous high-dimensional data reduction, our analysis identified systemic immune-inflammatory indices, immuno-nutritional surrogates, and specific local sonographic features as the core drivers of early hemodynamic deterioration. The Random Forest model demonstrated favorable discrimination and decision-analytic performance across both the internal and independent external validation cohorts. Furthermore, by integrating the SHapley Additive exPlanations framework and deploying the algorithm as a publicly accessible web-based calculator, we transformed complex multidimensional algorithms into a transparent, point-of-care clinical tool. This provides a potential framework for individualized risk stratification at hospital admission. Whether use of the calculator can facilitate earlier resuscitation, prioritize source control, or improve clinical outcomes requires prospective impact evaluation.

The main finding of our study was that the Systemic Immune-Inflammation Index and the C-Reactive Protein to Albumin Ratio made substantial contributions to the model’s prediction. From an immunological perspective, the transition from localized appendiceal inflammation to systemic septic shock is driven by a dysregulated host immune response (Bacârea et al., 2024). The Systemic Immune-Inflammation Index comprehensively captures the delicate balance among aggressive innate immunity, adaptive immune depletion, and coagulation cascade activation (Kollu et al., 2025). During early sepsis, peripheral neutrophils are massively recruited and activated, releasing reactive oxygen species and forming neutrophil extracellular traps (Liang and Su, 2024). Concurrently, platelets act as critical immune effectors by interacting with neutrophils to promote microvascular thrombosis, a hallmark of septic shock pathogenesis (Baran et al., 2025). Meanwhile, sepsis induces rapid apoptosis of lymphocytes, leading to a state of profound adaptive immunosuppression. Therefore, an elevated Systemic Immune-Inflammation Index effectively mirrors the lethal combination of hyperinflammation, microcirculatory dysfunction, and immune paralysis (Qiu et al., 2024). Similarly, the C-Reactive Protein to Albumin Ratio quantifies the acute-phase response against hepatic synthetic function, serving as a highly sensitive barometer for the intensity of the systemic inflammatory cytokine storm (Akboga et al., 2023; Hasanah et al., 2023).

Beyond classical inflammation, our model highlights the indispensable role of metabolic and immuno-nutritional crosstalk, specifically represented by the Glucose-to-Lymphocyte Ratio and the Prognostic Nutritional Index. Septic shock fundamentally involves massive cellular metabolic reprogramming (Lazzaro et al., 2022). An elevated Glucose-to-Lymphocyte Ratio reflects severe stress hyperglycemia driven by counter-regulatory hormones and insulin resistance, coupled with infection-induced lymphopenia (Schuetz et al., 2011). High circulating glucose concentrations further impair neutrophil phagocytosis and macrophage function, exacerbating immune dysregulation (Joffre and Hellman, 2021). Conversely, the Prognostic Nutritional Index serves as a critical indicator of the baseline immunological and nutritional reserve of the host (Wiedermann, 2021). Hypoalbuminemia in acute sepsis is not merely a sign of malnutrition but reflects severe systemic capillary leak syndrome and endothelial dysfunction (Xie et al., 2022). Patients with a diminished Prognostic Nutritional Index lack the necessary physiological buffer to endure severe septic insults, thus exhibiting a significantly higher probability of rapid clinical deterioration (Lou et al., 2025).

Crucially, our machine learning model bridges the gap between these systemic blood profiles and localized anatomical pathology. The SHAP analysis revealed that specific sonographic features, particularly the loss of the submucosal echogenic layer and the presence of periappendiceal fluid, act as potent independent predictors of septic shock. The submucosa is rich in vascular and lymphatic networks. The sonographic obliteration of this layer strongly indicates severe transmural ischemia, purulent micro-abscess formation, and impending or established gangrene (Ţocu et al., 2025). This irreversible structural destruction implies the complete failure of the local mucosal barrier. Such barrier breakdown provides a direct anatomical gateway for massive quantities of intestinal flora and endotoxins to translocate into the portal and systemic circulation (Bhatnagar et al., 2021; Martinez et al., 2023). These findings suggest that local sonographic abnormalities and systemic inflammatory indices may provide complementary predictive information. However, the present analysis does not establish a causal pathway, biological interaction, or synergistic effect between these features (Wang et al., 2023). The contribution of systemic inflammatory markers and local disease-related features to the model predictions may reflect complementary clinical information. However, because formal interaction analyses were not performed, these findings should not be interpreted as evidence of biological synergy or a causal relationship.

Despite the use of multiple complementary analytical procedures, several limitations of this study must be acknowledged. First, the retrospective design inherently carries a potential risk of selection bias, although the strict consecutive enrollment and multicenter validation substantially mitigate this concern. Second, the predictive model relies on baseline parameters obtained strictly upon hospital admission. The dynamic trajectories of these immune-inflammatory indices over time were not captured, which might provide additional prognostic insights during the initial resuscitation phase. Third, while we comprehensively evaluated classical systemic inflammatory markers, specialized immunological cytokines such as Interleukin-6 or Tumor Necrosis Factor-alpha were not routinely measured in the emergency setting and were therefore unavailable for inclusion. Future prospective studies incorporating dynamic biomarker monitoring and advanced multi-omics profiling are warranted to further refine this predictive framework. The primary analysis used treatment-specific observation windows, which may have introduced ascertainment bias. However, the consistent findings obtained using a uniform 48-hour prediction horizon support the robustness of the main results. Although class weighting and stratified sampling were used to address the relatively low prevalence of septic shock, the limited number of positive events may still have contributed to uncertainty in the performance estimates, particularly in the external validation cohort. Further studies with larger numbers of septic shock events are warranted.

## Conclusion

In conclusion, this multicenter study successfully developed and validated a highly accurate and interpretable machine learning model for predicting early septic shock in patients with acute appendicitis. By integrating systemic immune-nutritional indices with localized sonographic morphological features, the best-performing algorithm may serve as a useful adjunct for early risk stratification. To facilitate direct point-of-care clinical application, this comprehensive predictive model has been deployed as a freely accessible web-based calculator at https://medicalinformaticsshinyposter.shinyapps.io/shock-infection-predictor/. This transparent digital tool may support personalized risk assessment and clinical decision-making. Prospective implementation studies are needed to determine whether its use can accelerate resuscitation, optimize clinical management, or improve patient outcomes.

## Data Availability

The raw data supporting the conclusions of this article will be made available by the authors, without undue reservation.

## Supplementary material

Supplementary Figure 1 Participant screening and cohort allocation. The flow diagram shows the numbers of patients screened, excluded according to the prespecified criteria, and included in the primary and external validation cohorts.

Supplementary Figure 2 Decision curve analysis of the prediction models. The Random Forest (RF) model (cyan) provides a consistently higher net clinical benefit compared to the qSOFA (orange) and Alvarado (green) scores across almost all threshold probabilities. The grey slanted line represents “Treat All, “ and the horizontal line represents “Treat None.
