# Supplementary material for: Systemic immune dysregulation and local barrier failure as predictors of septic shock in acute appendicitis: a large-scale multicenter interpretable machine learning study
Source: Front Cell Infect Microbiol. 2026 Oct 16;16:1935756. doi: 10.3389/fcimb.2026.1935756 (PMC13719369; doi:10.3389/fcimb.2026.1935756)
Supplement: Supplementary file 1 [file Table1.docx]

**Supplementary Table S1.** Sensitivity analysis using a uniform 48-hour prediction horizon

| **Cohort** | **Total, n** | **Septic shock within 48 h, n (%)** | **No septic shock within 48 h, n (%)** | **AUC (95% CI)** | **Sensitivity** | **Specificity** | **PPV** | **NPV** |
| --- | --- | --- | --- | --- | --- | --- | --- | --- |
| Training cohort | 1,400 | 70 (5.0%) | 1,330 (95.0%) | 0.902 (0.878–0.926) | 0.843 | 0.840 | 0.217 | 0.982 |
| Internal testing cohort | 600 | 30 (5.0%) | 570 (95.0%) | 0.886 (0.838–0.934) | 0.800 | 0.819 | 0.189 | 0.952 |
| External validation cohort | 1,000 | 50 (5.0%) | 950 (95.0%) | 0.878 (0.839–0.917) | 0.780 | 0.831 | 0.195 | 0.953 |
