# Supplementary material for: Systemic immune dysregulation and local barrier failure as predictors of septic shock in acute appendicitis: a large-scale multicenter interpretable machine learning study
Source: Front Cell Infect Microbiol. 2026 Oct 16;16:1935756. doi: 10.3389/fcimb.2026.1935756 (PMC13719369; doi:10.3389/fcimb.2026.1935756)
Supplement: Supplementary file 2 [file Table2.docx]

**Supplementary Table S2.**Performance of the Random Forest model considering class imbalance

| **Cohort** | **Total, n** | **Septic shock, n (%)** | **ROC-AUC (95% CI)** | **PR-AUC** | **Sensitivity** | **Specificity** | **PPV** | **NPV** |
| --- | --- | --- | --- | --- | --- | --- | --- | --- |
| Training cohort | 1,400 | 84 (6.0%) | 0.914 (0.893–0.941) | 0.421 | 0.845 | 0.842 | 0.248 | 0.987 |
| Internal testing cohort | 600 | 37 (6.2%) | 0.829 ( 0.790-0.868) | 0.356 | 0.811 | 0.824 | 0.232 | 0.958 |
| External validation cohort | 1,000 | 58 (5.8%) | 0.812 (0.772-0.853) | 0.331 | 0.793 | 0.819 | 0.235 | 0.956 |
