# Supplementary material for: Systemic immune dysregulation and local barrier failure as predictors of septic shock in acute appendicitis: a large-scale multicenter interpretable machine learning study
Source: Front Cell Infect Microbiol. 2026 Oct 16;16:1935756. doi: 10.3389/fcimb.2026.1935756 (PMC13719369; doi:10.3389/fcimb.2026.1935756)
Supplement: Supplementary file 3 [file Table3.docx]

**Supplementary Table 3.** Calibration performance in the training, internal testing, and external validation cohorts

| **Cohort** | **Calibration intercept (95% CI)** | **Calibration slope (95% CI)** | **Brier score (95% CI)** |
| --- | --- | --- | --- |
| Training cohort | 0.00 (-0.26 to 0.26) | 1.00 (0.82 to 1.18) | 0.156 (0.134 to 0.167) |
| Internal testing cohort | -0.00 (-0.22 to 0.22) | 1.00 (0.80 to 1.20) | 0.196 (0.178 to 0.221) |
| External validation cohorts | -0.00 (-0.22 to 0.22) | 1.00 (0.80 to 1.20) | 0.183 (0.146 to 0.287) |
