# Supplementary material for: Systemic immune dysregulation and local barrier failure as predictors of septic shock in acute appendicitis: a large-scale multicenter interpretable machine learning study
Source: Front Cell Infect Microbiol. 2026 Oct 16;16:1935756. doi: 10.3389/fcimb.2026.1935756 (PMC13719369; doi:10.3389/fcimb.2026.1935756)
Supplement: Supplementary file 4 [file Table4.docx]

**Supplementary Table S4.** Performance comparison of the final Random Forest model and established clinical scores across three cohorts

| **Cohort / Model** | **ROC-AUC (95% CI)** | **P value (vs. RF)*** | **Brier score (95% CI)**** |
| --- | --- | --- | --- |
| **Training cohort (n=1400)** |  |  |  |
| Random Forest model | 0.914 (0.893–0.941) | Reference | 0.156 (0.134–0.167) |
| qSOFA score | 0.725 (0.672–0.778) | < 0.001 | 0.231 (0.211–0.251) |
| Alvarado score | 0.612 (0.558–0.666) | < 0.001 | 0.264 (0.245–0.283) |
| **Internal testing cohort (n=600)** |  |  |  |
| Random Forest model | 0.829 (0.790–0.868) | Reference | 0.196 (0.178–0.221) |
| qSOFA score | 0.685 (0.615–0.755) | < 0.001 | 0.252 (0.225–0.279) |
| Alvarado score | 0.584 (0.512–0.656) | < 0.001 | 0.285 (0.258–0.312) |
| **External validation cohort (n=1000)** |  |  |  |
| Random Forest model | 0.812 (0.772–0.853) | Reference | 0.183 (0.146–0.287) |
| qSOFA score | 0.671 (0.608–0.734) | < 0.001 | 0.268 (0.230–0.306) |
| Alvarado score | 0.575 (0.510–0.640) | < 0.001 | 0.291 (0.255–0.327) |
